# Enhanced performance of photonic crystal GaN light-emitting diodes with graphene transparent electrodes

**DOI:** 10.1186/s11671-015-0797-3

**Published:** 2015-03-01

**Authors:** Hai-Liang Ge, Chen Xu, Kun Xu, Meng Xun, Jun Wang, Jie Liu

**Affiliations:** The Key Laboratory of Optoelectronics Technology, Ministry of Education, Beijing University of Technology, Beijing, 100124 People’s Republic of China

**Keywords:** GaN-based light-emitting diodes, Graphene, Photonic crystal

## Abstract

The two-dimensional (2D) triangle lattice air hole photonic crystal (PC) GaN-based light-emitting diodes (LED) with double-layer graphene transparent electrodes (DGTE) have been produced. The current spreading effect of the double-layer graphene (GR) on the surface of the PC structure of the LED has been researched. Specially, we found that the part of the graphene suspending over the air hole of the PC structure was of much higher conductivity, which reduced the average sheet resistance of the graphene transparent conducting electrode and improved the current spreading of the PC LED. Therefore, the work voltage of the DGTE-PC LED was obviously decreased, and the output power was greatly enhanced. The COMSOL software was used to simulate the current density distribution of the samples. The results show that the etching of PC structure results in the degradation of the current spreading and that the graphene transparent conducting electrode can offer an uniform current spreading in the DGTE-PC LED.

**PACS:** 85.60.Jb; 68.65.Pq; 42.70.Qs

## Background

The GaN-based light-emitting diodes (LED) have recently attracted considerable interest because of advantages in low energy consumption, high brightness, and long lifetime. They have been widely used in various applications, such as full-color displays, general lighting, etc. [1-3]. However, the low light extraction efficiency (η_extr_) is a primary obstacle to the realization of higher brightness GaN LED [4-6]. In order to enhance the η_extr_, several approaches have been proposed, including patterned sapphire substrate, surface roughening, and PC structure [2-5]. With spatially periodic refractive index, the PC structure is one of the most promising approaches to efficiently reduce the loss caused by the total internal reflection at the interface of the GaN and the air [3-7]. In the previous works, the 2D PC structure was formed on the p-GaN layer or the indium tin oxide (ITO) layer of the LED, and the former showed better performance in light output power [8-11]. However, the forming of the PC structure may lead to the increase of the series resistance and the degradation of the work voltage of the LED, whether it was formed on the p-GaN layer or on the ITO layer.

So far, ITO has been used as the typical material for transparent electrodes. However, it is not appropriate for ultraviolet (UV) GaN LED [12,13]. In addition, the price of the ITO has been increasing for scarce of indium [14-17]. What’s more, ITO is unstable in chemical solutions [12,15,16]. Graphene, a 2D monolayer of carbon atoms, has recently attracted tremendous attention for its excellent optical, mechanical, and electrical properties, such as high transparency in the UV region, ultra-fast mobility, high thermal conductivity, and high mechanical strength [12-18]. It may be used as an alternative to the ITO in LED. Unfortunately, the sheet resistance of a single layer of graphene grown by chemical vapor deposition (CVD) is about as high as 500 to 1,000 Ω/□ [15,19]. Though the conductivity of graphene can be improved by doping, the doped graphene might be unstable and less transparent [15,16,20]. On the other hand, the carrier mobility in suspended graphene is much higher than that of graphene on substrates [15,21,22]. The sheet resistance of the suspended few layer graphene grown by CVD can reach 10 Ω/□ [15,23,22], which is lower than that of the ITO films applied in GaN LED. There have been reports about the suspended graphene used in optoelectronic devices [5,11,15], but few concerned with the graphene as the transparent conducting electrode in PC LED.

In this paper, we produced the PC LED with graphene transparent conducting electrode. The part of the graphene suspending over the air hole of the PC structure shows much higher conductivity and thus decreases the average sheet resistance of the graphene electrode and improves the current spreading, which is benefit to enhance the η_extr_ of PC LED.

## Methods

**Figure 1:**
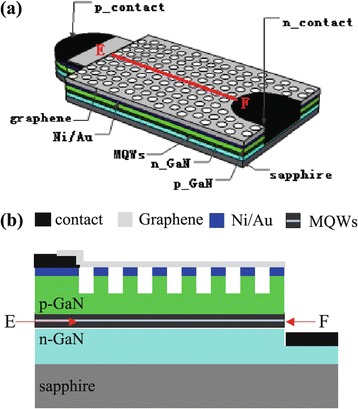
**DGTE-PC LED images. (a)** Schematic diagrams of the DGTE-PC LED; **(b)** schematic cross-section diagrams of the DGTE-PC LED.

Figure 1 shows the schematic diagrams of the DGTE-PC LED. It is consisted of the following layers: the DGTE layer, the p-GaN top cladding layer with PC structure, the multiple-quantum-well (MQW) active layer, the n-GaN bottom cladding layer, and the sapphire substrates. To form the DGTE-PC structure, the mesa process for n-electrode was executed. The p-GaN layer was etched by ICP to form triangle lattice air hole PC structure. Then, the Ti/Au layer was deposited as n- and p-pad electrodes, respectively. Finally, the single-layer graphene grown by CVD was transferred to the surface of the LED for two times, to form the transparent electrodes. The Ni/Au thin layer was inserted between the graphene and the p-GaN to get a better ohmic contact [24-26]. The sheet resistances of the mono- and two-layer graphene are about ~600 Ω/□ and ~300 Ω/□, respectively.

**Figure 2:**
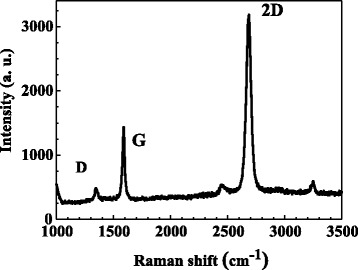
**Raman spectrum of graphene used for the DGTE-PC LED device.**

Figure 2 shows the Raman spectrum of the graphene film used for the DGTE-PC LED. Three prominent peaks in the Raman spectrum of the graphene film were observed: 2D, G, and D peaks, which are related to the quality of the graphene film [17]. The presence of G and 2D peaks is at ~1,600 and ~2,700 cm^−1^, respectively. The 2D intensity is over twice higher than that of G intensity. The lower D peak reveals the good quality of the graphene [17].

**Figure 3:**
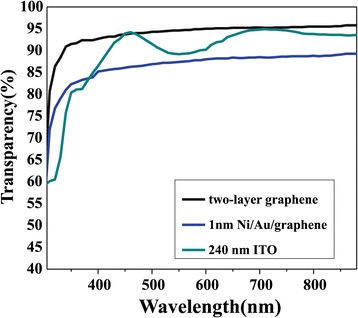
**Transmission spectra.**

Figure 3 exhibits the transmission spectra of the graphene conducting electrodes. The two-layer graphene film exhibits a transparency of nearly 95% to visible light, which is comparable to that of the 240-nm-thick ITO around 460 nm. The transmittance of the Ni/Au/graphene hybrid structure in the experiment is nearly 85% to visible light.

The hole radius and period of the PC structure are 750 and 3,000 nm, respectively. The etched depth of air hole is about 115 nm. Five kinds of LED samples were made. The conventional LED (without PC structure) with double-layer graphene electrode was signed as A2#; the conventional LED without graphene electrode was signed as A0#; the DGTE-PC LED sample was signed as B2#; the PC LED with the single-layer graphene electrode was signed as B1#; the PC LED without graphene electrode was signed as B0#.

## Results and discussion

**Figure 4:**
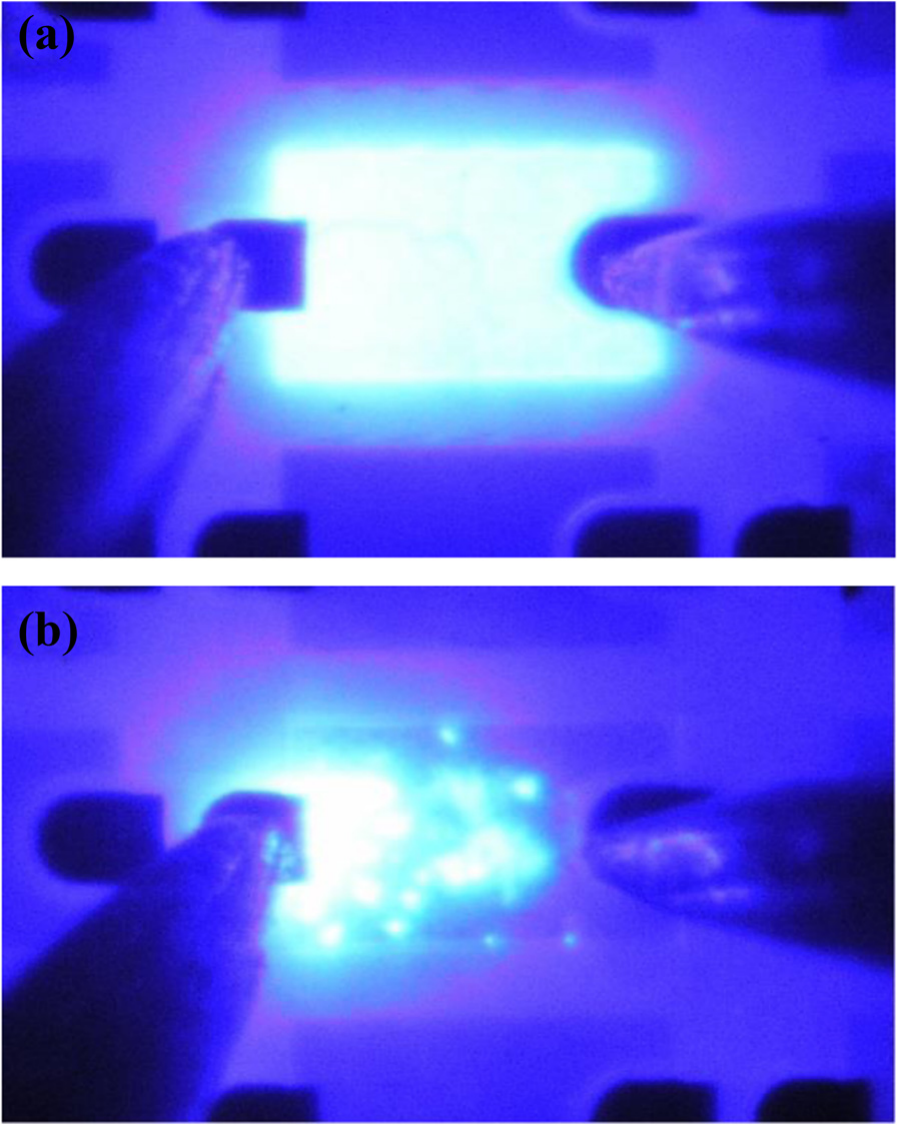
**The light emission patterns of B2# and B0#. (a)** Photographic images of the B2# sample; **(b)** photographic images of the B0# sample.

Figure 4 shows the light emission patterns of B2# and B0#, under injection current of I = 20 mA. The light emission of the B0# sample is rather uneven, resulting from the lack of the graphene transparent conducting electrode. In contrast, the B2# sample shows uniform light emitting over the entire LED surface, indicating that the double-layer graphene film offers a sufficient current spreading.

**Figure 5:**
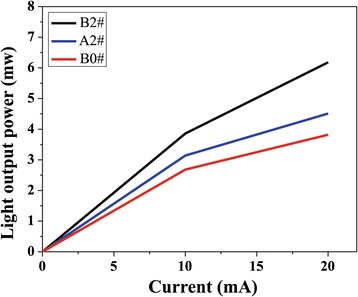
**The output power of LEDs.**

Figure 5 shows the light output-current characteristics of B2#, A2#, and B0# at I = 20 mA. The output power of the B2# is 6.1 mW, about 60% higher than that of B0# (3.8 mW), owing to the uniform current spreading, the wider lighting area, and the lower series resistance of the DGTE-PC LED.

**Figure 6:**
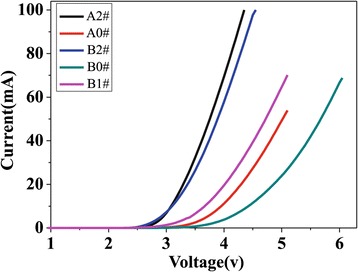
**The I to V characteristics of the LEDs.**

Figure 6 shows the current–voltage (I to V) characteristics of the five LED samples at an input current of 20 mA. The forward voltage of the A2#, A0#, B2#, B1#, and B0# samples is 3.3, 4.3, 3.4, 4, and 4.9 V, respectively. The work voltage of the A2# is 1 V lower than that of A0#, while the work voltage of the B2# sample is 1.5 V lower than that of B0#. This indicates that the double-layer graphene can greatly reduce the series resistance of LED, especially for PC LED due to improving the current spreading. The work voltage of the B2# sample is 0.6 V lower than that of B1#, showing the better effect of double-layer graphene. The work voltage of the B0# sample is 0.6 V higher than that of A0#, indicating the degraded current spreading in the PC LED because of a surface defect induced by ICP etching [15,27,28]. A parameter K is introduced to represent the overall degradation level of the forward I to V characteristics of the PC LED, compared with the conventional LED. K = △V/V_conventional_, where the △V represents the work voltage difference between the PC LED and conventional LED and the V_conventional_ stands for the work voltage of the conventional LED at I = 20 mA, respectively. The smaller the K is, the weaker the degradation of the forward I to V characteristics of the PC LED is. According to Figure 6, for B2# and A2# samples, K = 0.1/3.3 = 0.03. Kim Dong Ho and Kim Tae Sun observed the degradation of the forward I to V characteristic of PC LED due to etching PC structure, and they used some other transparent electrodes (ITO et al.) to improve the current spreading of the PC LEDs [5,11]. However, K = 0.12 and K = 0.15 can be obtained from their results, which is much higher than that of our results. Therefore, the advantage of the double-layer graphene electrode for the PC LED is obvious.

**Figure 7:**
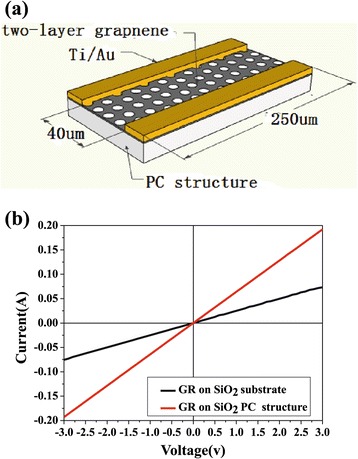
**Images of SiO**_2_ **PC structure and SiO**_2_ **PC and flat SiO**_2_ **substrate. (a)** The structure used to measure graphene sheet resistance on SiO_2_ PC structure. **(b)** The I to V curves of the two-layer graphene on SiO_2_ PC and on flat SiO_2_ substrate.

The part of the graphene suspending over the air hole of the PC structure takes an important role in improving the output power and reducing the work voltage of the PC LED. The conductivity of the suspended graphene is much higher than that of the graphene on planar substrate, due to the reduced carrier scattering from the substrate [15,21,22]. In order to investigate the conductivity of the suspended graphene, we measure the I to V characteristics of the double-layer graphene film on SiO_2_ PC structure (exactly the same as those used in the DGTE-PC LED), shown in Figure 7a and on SiO_2_ planar substrate, respectively. The Ti/Au electrode is annealed in N_2_ atmosphere for 2 min at 400°C to provide good ohmic contact. From test results, shown in Figure 7(b), the slope of the I to V characteristics of graphene on the SiO_2_ PC structure is 2.8 times as high as that on the planar substrate. The sheet resistance of graphene on the planar SiO_2_ substrate is about 300 Ω/□ and the average sheet resistance of graphene on the PC structure is about 107 Ω/□, which is similar to other reports [15,22].

**Figure 8:**
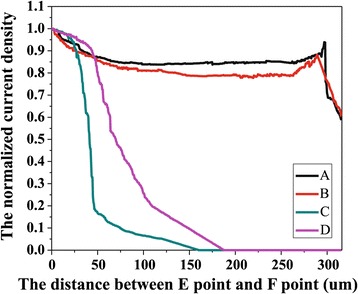
**The image of the normalized current density along the E-F line.** A The normalized current density of the LED with the graphene electrode of 150 Ω/□; B that of the LED with the graphene electrode of 300 Ω/□; C that of the PC LED without graphene electrode; D that of the conventional LED without graphene.

To quantitatively describe the impact of graphene on the current spreading, the COMSOL software was used to simulate the current density distribution on the surface of the MQWs layer of the samples. We choose the current density along the typical E-F line (as shown in Figure 1) at the injection current of I = 20 mA, shown in Figure 8. We can find that the current spreading of the B0# sample is seriously degraded, compare with that of the A0# sample, showing the current spreading degradation due to the etching of PC structure. The current spreading of the LED is improved obviously when the graphene electrode is used. The curve of the current density of the LED with the graphene electrode of 150 Ω/□ is more flat, showing better current spreading than that of the LED with the graphene electrode of 300 Ω/□. The lower the sheet resistance of the graphene is, the better the current spreading is. Obviously, the suspended graphene over the air hole is beneficial for the current spreading. The tail of the curve may be caused by the agglomeration effect of the current. It can be seen that the simulation results and experimental results are consistent.

## Conclusions

In summary, we have fabricated the DGTE-PC LED to investigate the key role of the graphene transparent conducting electrode in current spreading. The average sheet resistance of the graphene transparent conducting electrode was reduced by two to three times owing to the much higher conductivity of the part of the graphene suspending over the air hole of the PC structure. Therefore, the work voltage of the DGTE-PC LED was obviously decreased, and the output power was enhanced by about 60%. The simulation was carried out to explain the experimental results.
